# Comparative study of nutritional composition and color traits of meats obtained from the horses and Korean native black pigs raised in Jeju Island

**DOI:** 10.5713/ajas.18.0159

**Published:** 2018-07-26

**Authors:** Pil-Nam Seong, Geun-Ho Kang, Soo-Huyn Cho, Beom-Young Park, Nam-Geon Park, Jin-Hyoung Kim, Hoa Van Ba

**Affiliations:** 1 Animal Products Development and Utilization Division, National Institute of Animal Science, Wanju 55365, Korea; 2 Subtropical Livestock Research Institute, National Institute of Animal Science, Jeju 63242, Korea

**Keywords:** Nutritional Composition, Mineral, Vitamin, Horse Meat, Korean Native Black Pork

## Abstract

**Objective:**

The present study aimed at comparing the nutritional composition and color traits between two meat types: Horse meat and pork from Korean native black pigs raised in Jeju Island.

**Methods:**

After slaughter 24 h, the *longissimus dorsi* samples were taken from left side carcasses of the 32-mo-old Jeju female breed horses and the 6-mo-old Korean native black pigs (n = 10 each). The samples were then placed into cool boxes containing ice packs and transported to the Laboratory of Meat Science where all visual fats and connective tissues were trimmed off and then the samples were ground. All the samples were analyzed for nutritional composition (proximate composition, minerals, vitamins, fatty acids, and amino acids) and color traits.

**Results:**

The horse meat contained significantly higher collagen, moisture and protein than the pork (p<0.05). The Jeju horse meat showed more desirable fatty acid profiles such as containing significantly lower saturated fatty acids (SFA), higher polyunsaturated fatty acids (PUFA) contents and PUFA/SFA ratios than the pork (p<0.05). Differences in concentrations of ten amino acids existed between the two meat types in which the horse meat had higher values for all these amino acids, total amino acids (20.33 g/100 g) and essential amino acids (10.06 g/100 g) than the pork (p<0.05). Also, the horse meat showed significantly higher concentrations of Fe (34.21 mg/100 g) and Cu (2.47 mg/100 g) than the pork (Fe, 17.42 mg/100 g and Cu, 1.51 mg/100 g) (p<0.05). All the vitamins detected showed statistical differences between the two meat types in which the horse meat had higher concentrations of vitamin B_1_ (25.19 mg/100 g), B_2_ (92.32 mg/100 g), B_3_ (2,115.51 mg/100 g), and B_5_ (67.13 mg/100 g) than the pork (p<0.05).

**Conclusion:**

Based on the results obtained in the study, it is concluded that the two meat types studied are rich in nutrients and the animal species strongly affected the nutritional values and color traits of the muscle tissues.

## INTRODUCTION

Jeju horse is also known as the Korean native breed. So far, the Jeju horses are quite popular and often raised in the Jeju Island (Korea) because of its ideal geographical circumstance and climatic environment. Horse farming began in the Jeju Island during the thirteenth century, and according to the recently-reported data the total Jeju horse population exceeds 600,000 heads [1]. In general, Jeju native horses are raised for three main purposes; racing, tourism services (riding) and meat production [2]. The average live weight of these horses finished with supplemented concentrate diet was about 320 to 350 kg at 28 to 32 mo-old [2]. In Korea, Jeju horse meat is quite popular and considered as a special food which is often consumed raw [1]. Up to present, scientific information regarding the genetics of the horse breed has been extensively published in scientific journals, textbooks and websites etc. [3]. However, few studies have also focused on evaluating the effects of post-harvest factors on the quality parameters of this meat type [2].

Similar to the Jeju horse, Korean native pigs, also referred to as Jeju black pigs (JBP), are also highly appreciated in terms of economic value. The breed is most commonly raised on Jeju Island and to a lesser extent in the Korean Peninsula. The JBP is a typical pig breed which is characterized by black glossy hair, big eyes, long straight noses, slower growth rate and lighter carcass weight than commercial pig breeds [4]. The meat from the JBP has received the most attention and preference by consumers in comparison to the other commercial imported pork breeds due to its greater juiciness, palatability and redness [5]. In comparison to pork of the other imported commercial breeds (e.g., Yorkshire and Landrace), the JBP shows higher quality characteristics [6].

So far, the information on nutritional composition of each red meat species (e.g., pork, beef, horse meat, and mutton) or the comparison of nutritional values between some meat types can be found in some review papers [7–10]. However, the data presented in these review papers were often collected from published research papers that used various examination/evaluation/analytic methods in different countries, which might result in large variations in the levels of nutritional composition.

Although a significant amount of the scientific information regarding the genetics, meat quality or factors affecting the quality characteristics of the Jeju horse meat and JBP has been reported, to the best of our knowledge there remains limited information regarding the nutritional composition of these two meat types. Furthermore, there has been no study focusing on the analysis and comparison of nutritional values between these two red meat types, since they are the popular animal breeds on Jeju Island and have become important contributors to the economy of the animal production sector in the country [1].

In order to provide the meat producers and processors as well as consumers with a detailed overview on the nutritional composition of the meat species, the present work focused on analyzing and comparing the chemical and nutritional composition as well as technological quality traits of two red meat types: horse meat and pork produced from the Jeju Island in Korea.

## MATERIALS AND METHODS

### Meat samples preparation

Jeju female crossbreed horses (32-mo-old, live weights of about 320 to 360 kg) and Jeju female black pigs (JBP, 6-mo-old with averaged live weights of about 60 to 70 kg) (n = 10 each) were obtained from local farms in Jeju province (Korea) and used in the present study. The growing conditions for the horses were same as those described in the previous study [2]. While, all the pigs were fed with the same concentrate diet after weaning until slaughter. After slaughter, the carcasses were chilled for 24 h in a chilling room (2°C±2°C). Following the chilling, the carcasses were transferred to a cutting room where the loin samples (*longissimus dorsi*, LD muscle from 1st to 13th thoracic vertebrae) were collected from the left carcass sides. The muscle samples were then placed in plastic bags and transported to the Laboratory of Meat Science, National Institute of Animal Science for analyses of chemical and nutritional composition. Prior to analyses, all visual fats and connective tissues were trimmed off the muscle samples and only the trimmed muscles were kept. The trimmed muscles were then ground using meat grinder.

### Proximate composition

The collagen, moisture, protein and fat contents were determined by using a Food Scan Lab 78810 (Foss Tecator Co., Ltd., Hillerod, Denmark), as described in previous study [2]. Briefly, approximately 180 to 200 g of homogenized sample (each) was distributed in the instrument’s round sample dish and loaded into the instrument’s sample chamber. Each sample was determined in triplicates.

### Mineral contents

Mineral contents were determined using the method of Matilainen and Tummavuori [11] as described in previous study [2]. Briefly, the ground meat samples (5 g each) were destroyed by dry ashing in a microwave ashing oven (MAS 7000, CEM Corp., Mathews, NC, USA) for 12 h with a final temperature of 600°C. The ash sample was dissolved in 10 mL of 37% HCl and distilled water (ratio, 1:1 v/v) solution before filtering through Whatman filter paper No. 1 (AEC scientific Co., Seoul, Korea). Minerals such as: Na (selected wavelength 588.9 nm), K (766.5 nm), Ca (422.7 nm), Mg (285 nm), P (470 nm), Fe (248.3 nm), and Zn (213.9 nm), Mn (279.5 nm), Cu (324.7 nm), and Cr (357.9 nm) were determined by atomic emission spectrophotometer ICP-OES (Spectro, Boschstr, Kleve, Germany). A calibration curve was made for each element, and calculated as mg/kg meat sample. Each sample was done in duplicates.

### Amino acid contents

The preparation of samples for the analysis of amino acids (AA) was done following the method of Aristoy and Toldra [12] with suitable modifications. Briefly, the samples (1 g each) were homogenized with 0.01 N HCl solutions for 1 min at 4°C. The homogenates were centrifuged at 10,000×g for 20 min at 4°C and then were filtered through 0.45 μm filters (Millipore Corp., Biedford, MA, USA). To avoid the oxidation of cysteine and methionine, 20 mL of performic acid was also added to each sample as a protective agent before the acid hydrolysis. The AA contents were determined by using an AA analyzer (model 8900A) equipped with an exchange column (4.6×60 mm) (Hitachi, Tokyo, Japan). The separation and detection of AAs were carried out using the method as described by Seong et al [2].

### Fatty acid profiles

To determine the fatty acid composition, the meat samples were extracted using a solvent mixture of chloroform: methanol (2:1, v/v) as described by Folch et al [13] and then the extract was methylated using the procedure of Morrison and Smith [14]. The fatty acids were separated on a capillary column (30 m×0.32 mm×0.25 μm film thickness) connected to a gas chromatography (GC, Model Star 3600, Varian Technologies, Palo Alto, CA, USA). The GC condition was set as follows: 250°C for injection port and 300°C for detector. The free fatty acids in samples were identified by comparing their retention times with those obtained from standard fatty acids. The results were expressed as relative percentages based on total peak area. Each sample was done in triplicates.

### Vitamin content

Vitamins (vitamin A, B_1_, B_2_, niacin, niacinamide, B_5_, B_6_, and E) in the meat samples were determined using a reversed-phase high performance liquid chromatography (Aglient 1200 series, Aglient, Santa Clara, CA, USA) according to the procedures of AOAC [15].

### Instrumental color measurement

The instrumental color was determined at 3 different areas on the freshly cut surface of each sample after 30 min blooming using a Minolta Chroma Meter CR-400 (Minolta Camera Co., Ltd., Osaka, Japan) that was standardized with a white plate (Y = 86.3, X = 0.3165, and y = 0.3242). Color was expressed according to the Commission International de l’Eclairage (CIE) system and reported as CIE L* (lightness), CIE a* (redness), CIE b* (yellowness), chroma, and hue angle (h°). The chroma and hue angle were calculated as (a*^2^+b*^2^)^0.5^ and tan^−1^ (b*/a*), respectively.

### Statistical analysis

Data was analyzed with the Statistic Analysis System (SAS) package (SAS Institute, Cary, NC, USA, 2007). The data were analyzed by using the general linear model procedure considering meat type as the main effect. Means were compared using Duncan’s multiple range test, with a significance of p<0.05.

## RESULTS AND DISCUSSION

### Proximate composition

The collagen, fat, protein and moisture contents in the meat types are presented in Table 1. Significant differences occurred in all the above parameters between the two meat types (p< 0.05). Particularly, the horse meat contained higher collagen, moisture and protein whereas, it had lower fat content in comparison to the pork from JBP. The differences in collagen contents could be related to differences in physical activity of the muscles between the animal species, while the result indicating higher protein in the horse meat could be due to its lower fat content. When compared to the obtained results, Sarries and Beriain [16] reported slightly lower levels of moisture (68.34%), protein (19.91%), and fat (3.01%) for the same muscle type of 16 mo-old horses. While, Juarez et al [17] reported greater level of moisture (72.32%) but lesser fat content (2.08%) for the LD muscle of 24 mo-old horse meat. These contrasting results may be attributed to the differences in the slaughter age and breeds used. Regarding the pork, van Heerden and Smith [18], and Pereira and Vicenta [9] reported that it had higher protein content (21% to 22%) but much lower fat content (3.9% to 4.7%) for the loin. The JBP is characterized by a high marbling level or high intramuscular fat content in comparison to the other pig breeds such as Landrace or Yorkshire [6]. Therefore, the lower protein content in the JBP as compared to those of other pig breeds could be due to its significantly higher fat content.

### Fatty acid compositions

The fatty acid composition in the both meat types are presented in Table 2. A total of fourteen fatty acids were detected in the meat samples. Of which, 10 fatty acids showed statistical differences between the two meat types (p<0.05). The horse meat presented significantly higher levels of C14:0, C16:1n-7, C18:3n-3, and C20:4n-6 while, higher levels of C16:0, C18:0, C18:1n-9, C18:1n-7, and C20:1n-9 was found in the JBP samples (p<0.05). The Jeju horse meat had significantly lower total saturated fatty acids (SFA) and higher total polyunsaturated fatty acids (PUFA) contents in comparison to those of the JBP (p<0.05). The results indicating the fatty acid contents differences could be attributed to the differences in feeding diet used or the digestion and absorption process which affects the lipids synthesis and accumulation in the muscle tissues between the two animal species [19].

Among the fatty acids detected, C18:0 (stearic acid) is known to have little effect on plasma cholesterol level, while C18:30n-3 and C20:5n-3 are well known to produce beneficial effects on human health [19]. When compared to the levels of individual fatty acids in the Jeju horse meat in the present study, Tateo et al [20] reported higher C18:0 (6.64%) and C18:3n-3 (4.53%) contents for the same muscle of 11-mo-old horses. These authors also reported higher total SFA content, lower monounsaturated fatty acids (MUFA) and similar PUFA contents. A study of Palo et al [21] reported higher SFA (44.49%) and lower MUFA (28.90%) for the LD muscle of horses slaughtered at 6–18-mo-old. Also, compared to the fatty acid results obtained on the pork samples, Reig et al [22] reported lower the C18:3n-3 content (0.22% to 1.14%) for pork meat from other breeds fed with various diets. Lower total PUFA content was also reported for pork from Iberian breed [23].

The n-3 PUFA content plays an important role in human diet as it affects the physiological processes, increasing the intake of n-3 fatty acids is associated with human health [24]. The outcome of the analysis revealed that the eicosapentaenoic acid (C20:50n-3) was only found in the horse meat and not JBP meat. The n-3 content was found significantly higher in the horse meat (3.86%) than in the JBP (0.28%). Furthermore, the Department of Health [25] recommended that the PUFA/SFA ratio for a healthy diet should be 0.40 or higher, while the ratio of n-6/n-3 fatty acids should be 4.0 or lower. The results showed that the PUFA/SFA ratios were significantly higher in the Jeju horse meat (0.63) than in the meat of Korean native black pigs (0.15) (p<0.05). The Jeju horse meat also presented a n-6/n-3 ratio (5.3) close to the recommended value of 4.0 whereas, the JBP samples showed a n-6/n-3 value higher than the recommended value. Comparing to the data obtained in the present study, Tateo et al [20] reported lower PUFA/SFA ratio (0.54) for the same muscle type of 11-mo-old horses. Regarding pork, Dazza et al [23] also reported an undesirable PUFA/SFA ratio (0.11) and n-6/n-3 ratio (18.3) for pork of an Iberian breed.

Comparing to the fatty acid compositions, PUFA/SFA and n-6/n-3 ratios of both meat types in the present study, Joo et al [26] reported the total SFA of 47.23%, 46.28%, and 51.20% for beef muscle tissues from Korean Hanwoo, American and Australian breeds. These authors also reported much lower the PUFA contents (3.38% to 3.72%), especially more undesirable ratios for the PUFA/SFA and n-6/n-3 ratios were also reported for these three beef breeds. In general, the Jeju horse meat in the present study showed healthier fatty acid profile, characterized by lower SFA, higher PUFA contents, and more favorable PUFA/SFA and n-6/n-3 ratios in comparison with those of the JBP.

### Amino acid compositions

The AA contents in the two meat types are presented in Table 3. It is known that meats from different animal species or the quality of protein sources are distinguished by their essential amino acids (EAA) content [9]. There are hundreds of different AAs which have been identified, however, only twenty are important for protein synthesis. Out of these twenty AAs, eight (e.g., isoleucine, leucine, lysine, methionine, tryptophan, threonine, valine, and phenylalanine) are essential for the human’s body normal physiological functions, and because they cannot synthesized they must be supplemented through diets. Under the currently analytic conditions and detection limit, a total of seventeen AAs were found from the both meat types. Significant differences in ten AAs (serine, glycine, alanine, isoleucine, leucine, tyrosine, phenylalanine, lysine, arginine, and proline) occurred between the two meat types in which the horse meat presented greater amounts for all these AAs in comparison to the pork of the JBP (p<0.05). Furthermore, the horse meat presented a significantly (p<0.05) greater amount of total AAs (20.33 g/100 g), total essential (10.06 g/100 g), and non-essential amino acids (NAA, 10.27 g/100 g) than the pork meat. However, no differences in the EAA/total AA and EAA/NAA ratios occurred between the two meat types (p>0.05). The results indicating the lower total AA and EAA contents in the pork samples could be attributed to its much higher total fat content (Table 1), since the meat of JBP is characterized by high degree of marbling [6]. In general, the concentrations of most AAs detected in the both meat types fell within the ranges for the horse meat [2,27]. When compared to the EAA/NAA ratios in both meat types in the present study, Franco et al [28] reported a lower ratio (0.81 to 0.83) for the same muscle type of 15-mo-old horses.

### Mineral contents

The concentrations of minerals detected in the both meat types are presented in Table 4. A total of eight different minerals was found from the samples, and they can be categorized as; macro-elements (e.g., Ca, P, Na, Mg, and K etc.): large quantities of which are required by human body, and microelements (e.g., Fe, Cu, and Zn): small quantities are required by humans. Among the found minerals the microelements group are the most important in the biological systems (e.g., enzymatic system and structural functions etc.) and are vital for maintaining human health [29]. The insufficient intake of these microelements can cause symptoms of nutritional deficiency [30]. The results revealed that all the minerals showed significant differences between the two meat types studied (p<0.05). Particularly, the horse meat had higher amounts of Fe (34.21 mg/100 g) and Cu (2.47 mg/100 g) than the pork of JBP (Fe, 17.42 mg/100 g and Cu, 1.51 mg/100 g) (p<0.05). While, the meat of JBP presented significantly greater amounts of all the macro-elements such as Ca, Na, Mg, and K than the horse meat (p<0.05).

It is known that red meats are the excellent sources of Fe which is one of the vital minerals needed for the optimum function of blood; Fe deficiency causes anemia, especially in pregnant women and children [29]. Comparing to the Fe contents present in the both meat types in the present study, Williams [10], Pereira and Vicenta [9], and Cabrera and Saadoun [7] reported lesser amounts of Fe (0.9 to 4.6 mg/100 g) for the LD muscle of other meat species such as beef (1.5 to 3.3 mg/100 g) and mutton (1.7 mg/100 g) or chicken breast (1 mg/100 g), duck meat (2.4 mg/100 g), and lamb etc. Also, when compared to the Fe concentrations reported for horse meat (2.65 to 3.9 mg/100 g) by Lorenzo et al [27] and pork meat (0.6 to 1.3 mg/100 g) by Pereira and Vicenta [9] for the other breeds, the present result’s results showed greater amounts for the pork of JBP.

Zn is one of the important trace elements which participate in the activity of hundreds of enzymes; an insufficient intake of Zn may cause many health problems such as anemia, poor growth, DNA damages etc. [31]. Red meats are considered as the richest sources of bioactive Zn, when compared to the Zn contents in the both meat types in the present study, Cabrera and Saadoun [7] and Williams [10] reported lesser amounts (0.5 to 8 mg/100 g) for beef, lamb, chicken, and duck etc. The Zn content in the JBP also presented several times higher compared to the levels (1.6 to 2.7 mg/100 g) of pork from other breeds [9]. According to the Dietary Reference Intakes (the highest level of minerals intake that does not cause any risk of health effects) for Fe (45 mg/d) and Zn (40 mg/d) for adults [29], thus a consuming 100 g of Jeju horse meat or Jeju black pork would supply 76.02% and 38.71% of Fe, and 117.60% and 96.85% of Zn, respectively. Based on the results obtained in the present study and literatures, it may be said that species and breeds have strong effects on the minerals content.

### Vitamins content

The concentrations of vitamins found in the both meat types are presented in Table 5. Among eight different vitamins found, four (vitamin B_1_, B_3_, B_3_, and B_5_) were detected on the both meat types. While, the vitamin A was not found in the horse meat and the three vitamins (niacinamide, vitamin B_6_, and E) were not found in the meat of JBP, probably be due to their concentrations being lower than the detection limits of the analytic conditions in the present study.

The results of statistical analysis revealed that significant differences in amounts of all the detected vitamins existed between the two meat types (p<0.05). Particularly, the horse meat had greater amounts of vitamin B_1_ (25.19 mg/100 g), B_2_ (92.32 mg/100 g), B_3_ (2,115.51 mg/100 g), and B_5_ (67.13 mg/100 g) in comparison to the meat of JBP (p<0.05). These contrasting results could be explained by the animal species differences. Among the detected vitamins which may be classified into: the water-soluble group (vitamins B) and fat-soluble group (vitamin A and E). It is well recognized that vitamins are essential for maintaining the normal function and metabolic reactions in the body. Particularly, vitamin E acts as a distinctive antioxidant compound in the body; together with vitamin A is involved in various physiological process (gene expression and immune system), growth and development while, the vitamin B group plays important roles such as: metabolism of AAs, hydrogen-transferring process and nervous system etc. [31]. When compared to the present study’s results, Williams [10] reported the vitamin B_1_ and B_2_ contents (3 and 25 mg/100 g, respectively) for beef, which was lesser than the values found in the Jeju horse meat and JBP in the present study. Noticeably, when compared to the concentrations of some vitamins found in the Jeju black pork in the present work, van Heerden and Smith [18] reported much lower vitamin B_1_ (0.22 mg/100 g) and B_2_ (0.02 mg/100 g) contents for the pork loin samples of other breeds. Similarly, Cashman and Hayes [8] reported much lower amounts of vitamin A (trace amount), B_1_ (0.1 to 0.8 mg/100 g), B_2_ (0.1 to 0.4 mg/100 g), and B_3_ (0.1 to 0.8 mg/100 g) for various meat species (beef, lamb, and veal meat) and pork meat from other breeds. These obtained results suggest that the animal species and breeds strongly affect the amounts of the vitamins in the muscle tissues.

### Instrumental colors

Meat color is one of the most important factors that affect the appearance, attractiveness and meat purchasing decisions by consumers. Myoglobin is known as the main protein responsible for the meat color; also some proteins such as hemoglobin and cytochrome C contribute to the color formation of meats [32]. The results (Table 6) showed that the meat of JBP was lighter compared to the horse meat. Whereas, the horse meat presented significantly higher CIE a* and b* values, indicating that the horse meat was redder and more yellow than the pork meat (p<0.05). These obtained results could be related to the differences in chemical compositions between the two meat types for instance; higher protein and fat contents (Table 1) responsible for the redder and lighter colors in horse meat and pork meat, respectively. The horse meat also had higher Chroma value and presented lower hue angle value than the pork meat (p<0.05). The meat color traits are known to be affected by many factors such as; breed, gender, age or muscle types [2,27,32]. The results indicating the color differences between the meat two meat types could be related to the effects of animal species. When compared to the color traits of the same muscle of horses from other breeds at different slaughter ages [27,33], the Jeju horse meat studied had the similar L*-value, but it presented considerably higher the CIE a*-values. Similarly, the meat of JBP also had redder color (indicated by higher CIE a* value) as compared to the same muscle of pork from other breeds ([Yorkshire×Landrace]×Duroc) reported in literature [34]. Thus, from the results obtained in the present study and those reported in literatures, it may be said that the meats from Jeju horses and JBP had more attractive colors which could be due to their higher myoglobin contents than those in meat of other breeds.

## CONCLUSION

The present study, for the first time the major nutritional composition of the two main meat types produced in Jeju Island were analyzed and compared with each other as well as compared with those reported for the other meat breeds and species in literatures. The Jeju horse meat had higher collagen and protein contents as compared to those of the pork. The horse meat contained significantly lower SFAs, higher PUFAs contents, and had more desirable PUFA/SFA ratios than the pork. Additionally, the horse meat also showed greater amounts of total AAs and EAA than those of the pork. Higher concentrations of the trace elements such as; Fe and Cu, and higher contents of the vitamin B group such as B_1_, B_2_, B_3_, and B_5_ were found in the horse meat. It is also noted that the concentrations of the important nutrients (e.g., minerals and vitamins) in these two meat types produced in Jeju Island were considerably higher than those reported for the meats from other animal breeds and meat species published in the literature. It is concluded that Jeju horse meat and Jeju black pork both are rich in nutrients and the animal species strongly affected the nutritional contents in the muscle tissues.

## Figures and Tables

**Table 1:** Comparison of proximate compositions in *longissimus dorsi* muscle between Jeju crossbred horse meat and Jeju black pork

Items	Jeju crossbred horse meat	Jeju black pork
Collagen (%)	1.59±0.06^a^	1.24±0.09^b^
Fat (%)	5.84±0.69^b^	15.77±1.54^a^
Moisture (%)	70.17±0.44^a^	63.80±0.96^b^
Protein (%)	20.31±0.34^a^	19.08±0.33^b^

a,b Means in the same row with different letters are significantly different (p<0.05).

**Table 2:** Comparison of fatty acid compositions in *longissimus dorsi* muscle between Jeju crossbred horse meat and Jeju black pork

Items	Jeju crossbred horse meat	Jeju black pork
Myristic acid (C14:0)	3.67±0.84^a^	1.98±0.11^b^
Palmitic acid (C16:0)	29.15±1.34^b^	33.12±1.11^a^
Palmitoleic acid (C16:1n-7)	6.06±1.23^a^	3.98±0.3^b^
Stearic acid (C18:0)	4.63±1.01^b^	13.89±0.75^a^
Oleic acid (C18:1n-9)	32.76±1.88^b^	38.61±0.99^a^
(C18:1n-7)	0.04±0.01^b^	0.21±0.04^a^
Linoleic acid (C18:2n-6)	18.67±1.59^a^	6.86±1.74^b^
℘-Linoleic acid (C18:3n-6)	0.03±0.01^a^	0.04±0.01^a^
Linolenic acid (C18:3-n3)	3.6±1.14^a^	0.28±0.04^b^
Gondonic acid (C20:1n-9)	0.3±0.01^b^	0.76±0.07^a^
Arachidonic acid (C20:4n-6)	0.73±0.06^a^	0.21±0.08^b^
Eicosapentaenoic acid (C20:5n-3)	0.02±0.01	ND
Adrenic acid (C22:4n-6)	0.09±0.07^a^	0.07±0.02^a^
Docosahexaenoic acid (C22:6n-3)	0.36±0.24	ND
SFA	37.45±1.24^b^	48.99±1.59^a^
MUFA	39.17±0.85^b^	43.56±1.10^a^
PUFA	23.38±1.85^a^	7.45±1.85^b^
n-3	3.86±0.96^a^	0.28±0.04^b^
n-6	19.52±2.2^a^	7.17±1.82^b^
n-6/n-3	5.3±1.41^b^	25.57±3.84^a^
MUFA/SFA	1.05±0.03^a^	0.89±0.04^b^
PUFA/SFA	0.63±0.07^a^	0.15±0.04^b^

ND, not detected; SFA, saturated fatty acids; MUFA, monounsaturated fatty acids; PUFA, polyunsaturated fatty acids.

a,b Means in the same row with different letters are significantly different (p<0.05).

**Table 3:** Comparison of amino acid concentrations (g/100 g) in *longissimus dorsi* muscle between Jeju crossbred horse meat and Jeju black pork

Items	Jeju crossbred horse meat	Jeju black pork
Essential amino acids		
Histidine	0.89±0.06^a^	0.71±0.15^a^
Isoleucine	0.88±0.06^a^	0.68±0.11^b^
Leucine	1.78±0.09^a^	1.42±0.22^b^
Lysine	1.86±0.09^a^	1.49±0.26^b^
Phenylalanine	0.84±0.04^a^	0.66±0.10^b^
Threonine	0.97±0.05^a^	0.83±0.12^a^
Valine	0.95±0.07^a^	0.80±0.12^a^
Methionine	0.53±0.03^a^	0.51±0.07^a^
Arginine	1.36±0.08^a^	1.09±0.20^b^
∑Essential amino acids	10.06±0.05^a^	8.19±0.26^b^
Non-essential amino acids		
Glycine	1.12±0.11^a^	0.86±0.14^b^
Alanine	1.31±0.10^a^	1.04±0.16^b^
Asparagine	1.94±0.01^a^	1.66±0.24^a^
Tyrosine	0.71±0.05^a^	0.52±0.07^b^
Serine	0.88±0.04^a^	0.75±0.09^b^
Glutamic acid	3.13±0.14^a^	2.70±0.39^a^
Cysteine	0.24±0.02^a^	0.21±0.03^a^
Proline	0.96±0.01^a^	0.69±0.12^b^
∑Non-essential amino acids	10.27±0.03^a^	8.44±0.22^b^
∑Amino acids	20.33±1.13^a^	16.63±2.56^b^
EAA/AA ratio1)	0.49±0.01^a^	49.24±0.02^a^
EAA/NAA ratio2)	0.97±0.02^a^	0.97±0.04^a^

1) EA/AA, essential amino acid to total amino acid ratio.

2) EA/NAA, essential amino acid to non-essential amino acid ratio.

ab Means in the same row with different letters are significantly different (p<0.05).

**Table 4:** Comparison of mineral contents in *longissimus dorsi* muscle between Jeju crossbred horse meat and Jeju black pork

Items	Jeju crossbred horse meat	Jeju black pork
Macro-elements (mg/100 g)		
Na	564.75±47.12^b^	1,067.93±36.24^a^
Ca	64.74±1.23^b^	115.1±12.55^a^
P	2,175.97±212.49^b^	4,173.36±192.03^a^
Mg	196.37±28.37^b^	447.96±30.13^a^
K	2,711.86±336.98^b^	6,555.36±524.58^a^
Microelements (mg/100 g)		
Cu	2.47±0.15^a^	1.51±0.21^b^
Zn	47.04±1.99^a^	38.74±4.11^a^
Fe	34.21±2.75^a^	17.42±2.52^b^

a,b Means in the same row with different letters are significantly different (p<0.05).

**Table 5:** Comparison of vitamin contents in *longissimus dorsi* muscle between Jeju crossbred horse meat and Jeju black pork

Items	Jeju crossbred horse meat	Jeju black pork
Vitamin A (μg RE/100 g)	ND	32.49±1.56
Vitamin B_1_ (mg/100 g)	25.19±1.02^a^	4.29±0.24^b^
Vitamin B_2_ (mg/100 g)	92.32±2.81^a^	0.50±0.01^b^
Vitamin B_3_ (mg/100 g)	2,115.51±177.58^a^	4.31±0.06^b^
Niacinamide (mg/100 g)	183.04±67.15	ND
Vitamin B_5_ (mg/100 g)	67.13±3.18^a^	0.57±0.04^b^
Vitamin B_6_ (mg/100 g)	2.57±0.54	ND
Vitamin E (mg/100 g)	0.20±0.02	ND

ND, not detected.

a,b Means in the same row with different letters are significantly different (p<0.05).

**Table 6:** Comparison of instrumental color characteristics in *longissimus dorsi* muscle between Jeju crossbred horse meat and Jeju black pork

Items	Jeju crossbred horse meat	Jeju black pork
CIE L* (lightness)	38.60±0.90^b^	48.46±1.20^a^
CIE a* (redness)	22.86±0.31^a^	7.93±0.48^b^
CIE b* (yellowness)	10.88±0.39^a^	3.72±0.25^b^
Chroma	24.81±0.50^a^	8.79±0.48^b^
Hue angle	24.79±1.03^b^	25.36±1.68^a^

CIE, Commission International de l’Eclairage.

a,b Means in the same row with different letters are significantly different (p<0.05).
